# Safety-Catch Linkers for Solid-Phase Peptide Synthesis

**DOI:** 10.3390/molecules29071429

**Published:** 2024-03-22

**Authors:** Sikabwe Noki, Beatriz G. de la Torre, Fernando Albericio

**Affiliations:** 1 Peptide Science Laboratory, School of Chemistry and Physics, University of KwaZulu-Natal, Westville, Durban 4000, South Africa; sikabwenoki7@gmail.com; 2 KwaZulu-Natal Research Innovation and Sequencing Platform (KRISP), School of Laboratory Medicine and Medical Sciences, College of Health Sciences, University of KwaZulu-Natal, Durban 4041, South Africa; 3 CIBER-BBN, Networking Centre on Bioengineering, Biomaterials, and Nanomedicine, Department of Organic Chemistry, University of Barcelona, 08028 Barcelona, Spain

**Keywords:** safety-catch linkers (SCLs), oxidative SCLs, reductive acidolysis SCLs, SPPS, protecting groups

## Abstract

Solid-phase peptide synthesis (SPPS) is the preferred strategy for synthesizing most peptides for research purposes and on a multi-kilogram scale. One key to the success of SPPS is the continual evolution and improvement of the original method proposed by Merrifield. Over the years, this approach has been enhanced with the introduction of new solid supports, protecting groups for amino acids, coupling reagents, and other tools. One of these improvements is the use of the so-called “safety-catch” linkers/resins. The linker is understood as the moiety that links the peptide to the solid support and protects the C-terminal carboxylic group. The “safety-catch” concept relies on linkers that are totally stable under the conditions needed for both α-amino and side-chain deprotection that, at the end of synthesis, can be made labile to one of those conditions by a simple chemical reaction (e.g., an alkylation). This unique characteristic enables the simultaneous use of two primary protecting strategies: tert-butoxycarbonyl (Boc) and fluorenylmethoxycarbonyl (Fmoc). Ultimately, at the end of synthesis, either acids (which are incompatible with Boc) or bases (which are incompatible with Fmoc) can be employed to cleave the peptide from the resin. This review focuses on the most significant “safety-catch” linkers.

## 1. Introduction

**Figure 1:**
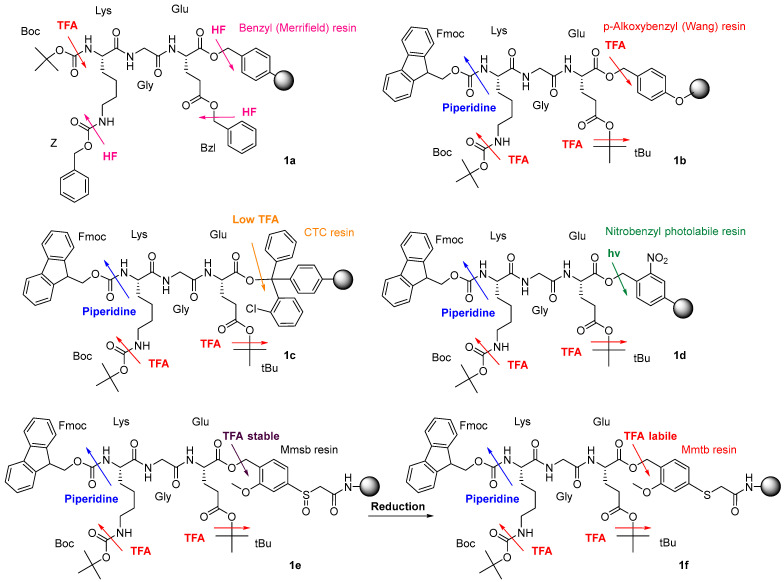
Different classes of protecting group schemes including the linker.

Solid-phase peptide synthesis (SPPS) is an innovative methodology that has revolutionized the field of peptide chemistry and peptide-based drug discovery. Developed by R.B. Merrifield in early 1963 [1], SPPS has since become a necessary tool for the effective synthesis of peptides. It was developed to overcome the limitations of traditional solution peptide synthesis [2]. This synthesis approach is much less time consuming, keeping suitable yields and purities of the final peptides. The need for SPPS arises from the difficulties of the manipulation of the intermediates in solution peptide synthesis. This traditional strategy involved repetitive isolation and, very often, purification of the intermediates, leading to a tedious workup, which very often translated into low yields [3]. Merrifield’s vision was to attach the peptide chain onto a solid support; in other words, to use a polymeric and solid C-terminal protecting group and avoid the isolation and purification of the intermediates. Furthermore, as the growing peptide was anchored to the solid support, excess reagents could be used because the unreacted reagents and the soluble side-products can easily be removed by simple washings [1]. As the growing peptide stays during all the synthetic processes anchored to the solid support, this can be considered a reactor and, therefore, is amenable to automation. SPPS has shown an important impact in multiple scientific disciplines, providing insights into a large number of biological processes [4,5,6]. However, SPPS has been pivotal in the drug discovery arena, allowing the speeding up of the first steps of the process (hit detection, hit to lead to candidate) and, at the end of the process, facilitates the production of peptides with more than 40 amino acids on a multi-kilogram scale with the sufficient purity to fulfill the requirements of the regulatory agencies and, therefore, to be used in humans [7,8]. SPPS can be defined as a proper combination of the protecting groups (PGs) and coupling reagents in a proper solvent. As mentioned earlier, the linkers are the protecting group of the C-carboxylic group. The linker should be totally stable during the elongation of the peptide and then should liberate the peptide with the best yield possible. Very often, the final cleavage will also remove the protecting groups of the side-chain of the amino acids. However, for other applications, the release of the peptide from the linker resin should keep those protecting groups rendering a protected peptide [9]. Considering the linker (or linker resins) versus the other protecting groups for α-amino and for the side-chain protecting groups, linkers can be classified into four categories: (i) Kinetic Fine-Tuning-Based; (ii) Bis-Orthogonal; (iii) Three-Orthogonal; and (iv) Safety-Catch. The best example of the first class is the Merrifield strategy, benzyl (Bzl) groups for the linker and the side-chain protecting groups, and tert-butyloxycarbonyl (Boc) for the α-amino (Figure 1a). The Boc and the Bzl groups are removed by the same mechanism, acidolysis, but through different kinetics. The Boc group is removed with trifluoroacetic acid (TFA) and the Bzl groups by anh. HF or trifluoromethanesulfonic acid (TFMSA) [10]. The best example of a bis-orthogonal category is the combination of p-methoxybenzyl (Wang) linker for SPPS, tert-butyl (tBu) for side-chain protecting groups, and fluorenylmethoxycarbonyl (Fmoc) for the α-amino group [11]. There are two different chemical mechanisms: acidolysis with TFA for the first and base through a β-elimination for the Fmoc (Figure 1b). Thus, we can remove ones in the presence of others and vice versa. Very important for this category is that the conditions can be forced; for instance, the Fmoc can be removed at high temperatures, keeping in place the rest of the groups. This is not possible in the kinetic fine-tuning-based systems, because removing the Boc at high temperatures or for long durations will detach the peptide from the resin. A variation of this class is when three different classes of protecting groups are present; one is independently orthogonal to the other two, which in turn are removed by the same chemical method but with different kinetics. The best example of this category is Cl-tritrylchloride (CTC) resin together with tBu side-chain protecting groups and Fmoc (Figure 1c). The latter is orthogonal to the remaining two, but the peptide is released from the CTC resin with dilute TFA (2%) solutions and the side-chain protecting groups with concentrated TFA. This means that the side-chain cannot be deprotected while the peptide is still anchored to the solid support [11]. A three-orthogonal scheme is then formed; for instance, a photolabile linker such as the nitroBzl together with tBu side-chain protecting groups and Fmoc (Figure 1d). The three different mechanisms are totally independent among them, and, therefore, it is possible to remove the protecting groups of the side-chain on the resin or cleave the protected peptide from the resin [12]. This ideal scheme allows for forcing the deprotection condition to increase the yields. The last category and object of this study is the safety-catch one.

The “safety-catch” concept relies on linkers totally stable under the conditions needed for both α-amino and side-chain deprotection that, at the end of synthesis, can be made labile to one of those conditions by a simple chemical reaction. For example, Figure 1e,f) show the uses of the pair 2-methoxy-4-methylsulfinylbenzyl alcohol (Mmsb) and 2-methoxy-4-methylthiobenzyl alcohol (Mmtb) [13]. Mmsb (Figure 1e) has a sulfinyl/sulfoxide electron-withdrawing group in the *para* position, which makes the ester bond that links the peptide to the resin stable under TFA conditions and, therefore, is compatible with Fmoc and Boc chemistry. However, at the end of the synthesis, a reductive treatment converts the sulfinyl/sulfoxide into a thio (Figure 1f), which is an electron-donating group, and, therefore, makes the ester bond cleavable with TFA.

### 1.1. Safety-Catch Linkers

As mentioned above, safety-catch linkers (SCLs) are stable during the elongation of the peptide chain usually to an acid and/or base, ensuring the attached peptides are securely held until intended release from the solid support [14,15]. This can take place after the modification of the linker, which makes it more labile under those conditions (acid or base) than before it was stable, etc. The fundamental principle of SCLs involves their inertness throughout the synthesis process, requiring conversion from a stable form to an activated, labile state before cleavage. An excellent application of SCLs is the synthesis of peptides with diverse functional groups at the C-terminal, including carboxylics, amides, thioesters, and hydrazides. These functional groups are valuable for a variety of downstream applications, such as native chemical ligations [16] and chemo-selective conjugations [17]. There are a number of SCLs that have been developed for years, each with its own application.

### 1.2. Kenner and Sulfonamide Safety-Catch Linker

**Scheme 1:**
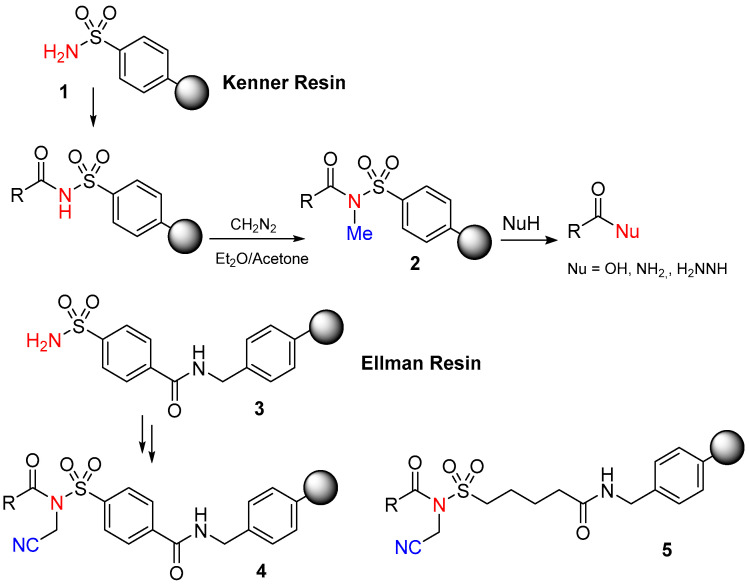
Kenner and Ellman safety-catch resins.

The initial carboxy anchor employed to illustrate the safety-catch principle was the sulfonamide resin (**1**) described by Kenner and co-workers (Scheme 1). [18] Acyl sulfonamides remain stable even in the presence of strong anhydrous acids, HBr/AcOH, as well as highly nucleophilic reagents and aqueous alkali. In this case, the stability persists because any basic attack ionizes the acidic SO_2_NH group (with a pKa~2.5). Their final cleavage involves activating a sulfonylacylamide group through the negative inductive effect of a nearby substituent, the sulfonyl. Thus, the *N*-alkylation turns the stable linker into its labile form in front of nucleophiles [18].

The initial safety-catch activation process of acyl-Kenner resin involved *N*-methylation with diazomethane (CH_2_N_2_) in diethylether (Et_2_O)/acetone, resulting in the formation of the labile *N*-methyl acyl sulfonamide **2**. This labile *N*-methyl acyl sulfonamide **2** can be cleaved in the presence of nucleophiles using alkaline hydrolysis (0.5 M NaOH), aminolysis (0.5 M NH_3_/dioxane), hydrazinolysis (methanolic hydrazine), and thiolysis.

The initial *N*-methyl acyl sulfonamide from Kenner’s resin has low reactivity, leading to poor cleavage yields with less nucleophilic amines like anilines and requiring excess reagent for successful cleavage with more nucleophilic alkylamines. To address this issue, Ellman and co-workers [19] have slightly modified the Kenner strategy (Ellman resin) (Scheme 1). First of all, they used a sulfamoylbenzamide **3**, which makes the NH more acidic, and then the activation step is carried with ICH_2_CN, enhancing the reactivity of linker **4** with various amines and nucleophiles [20].

**Scheme 2:**
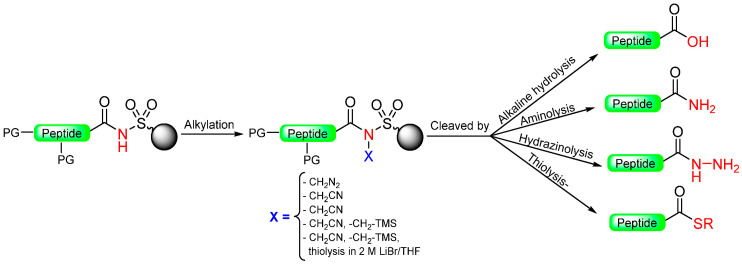
General acyl sulfonamide safety-catch linker, activation, and cleavage.

This method allows the use of limited amounts of even sterically hindered and non-basic amines, diversifying the final amide products. The linker is also compatible with common peptide-coupling reagents, enolate alkylation [19], and Suzuki reaction conditions [21]. However, there are limitations, such as incomplete alkylation of the acyl sulfonamide when carboxylic acids with an α-electron-withdrawing group are used (Scheme 1). This limitation has been overcome by using aliphatic sulfonamide linker **5** [22]. Comparative experiments demonstrated improved cleavage yields compared to the original aryl sulfonamide linker **1**. Nevertheless, challenges, such as incomplete and racemization-free acylation with protected amino acids due to the low nucleophilicity of the sulfonamide and the strong basic conditions required for the alkylation, still need to be addressed. A different method to activate *N*-acyl-sulfonamide nitrogen by using palladium (0) catalysis and allylation was proposed [23]. This generated a highly reactive intermediate that enables activation under gentle and neutral conditions, reducing the risk of racemization in the first protected amino acid [24] (Scheme 2). Although Kenner’s linker was first used with tert-butoxycarbonyl (Boc)-benzyl (Bzl) chemistry, it is perfectly compatible with fluorenyl methoxycarbonyl (Fmoc)-tert-butyl (tBu) chemistry.

### 1.3. Isonitrile/Benzamide Safety-Catch Linker

**Scheme 3:**
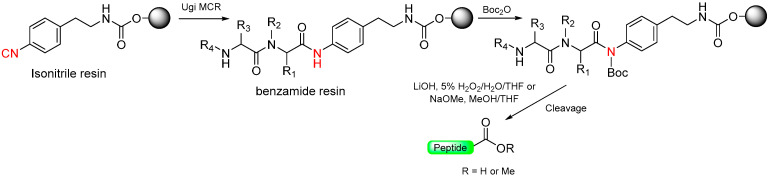
*N*-Protection with Boc safety-catch linkers.

A variant of the Kenner idea is the isonitrile/benzamide linker. The resin-bound isonitrile allows for construction of the peptide on the resin using a Ugi multicomponent reaction (MCR) [25,26]. At the end of the synthetic process, the benzamide resin is activated by Boc protection of the benzamide, which becomes susceptible to hydrolytic cleavage (aq. LiOH/H_2_O_2_) to produce peptide carboxylic acids, or alcoholic cleavage (MeONa/MeOH) to yield methyl ester peptides, respectively (Scheme 3) [26].

### 1.4. The Oxidative Safety-Catch Linkers

**Scheme 4:**
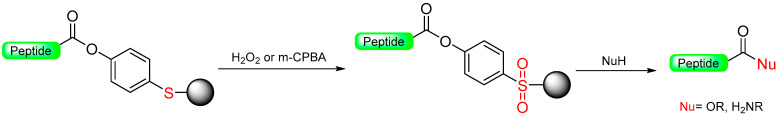
Marshall’s oxidative safety-catch linker.

Marshall et al. introduced the oxidative safety-catch linker, initially designed for protecting peptide fragments using Boc/Bzl chemistry [27]. They proposed a phenolsulfide resin (Scheme 4) conveniently prepared from chloromethylated polystyrene resin and p-mercaptophenol carbonate. The peptide chain was extended using the Boc/Bzl strategy. The oxidative activation of the linker was carried out using H_2_O_2_ to produce the sulfone linker. This facilitated cleavage through alkali hydrolysis and ammonolysis, leading to the release of the fully protected peptide [28]. A similar application of this phenolsulfide linker was reported, where m-CPBA was used to activate the linker to sulfone, and an internal aminolysis cleavage released the cyclic peptide [28]. Yager et al. [29] determined that oxidation of the linker is not necessary for the efficient cleavage of peptides because the phenol group is a suitable enough leaving group [30]. A significant challenge hindering the practical use of this linker is its own lability in front of the bases, which makes it not compatible with a Fmoc/tBu strategy. Furthermore, the oxidation process can also exclude delicate amino acids like Met and Cys [31].

**Scheme 5:**
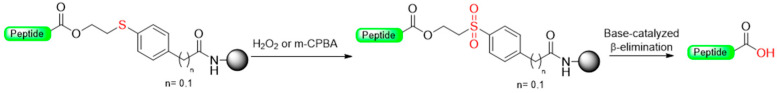
Base-catalyzed *β*-elimination safety-catch linker.

Our group [32] recently developed a base-catalyzed *β*-elimination safety-catch linker, 2-hydroxyethylthio acid derivatives (Scheme 5) [32]. The first protected amino acid forms an ester bond stable to bases such as secondary amines and acids such as trifluoroacetic acid (TFA) and, therefore, compatible with Fmoc and Boc chemistry. After the elongation of the peptide chain has taken place, the oxidation to the sulfone form will convert the ester bond labile secondary amines through a *β*-elimination reaction. The linker activation was conducted in SPPS using a multidetachable (double) linker strategy.

Schwyzer et al. developed a similar oxidative safety-catch linker that can be used for the synthesis of oligonucleotides [33]. Furthermore, Garcia-Echeverria [34] and Sowing and co-workers [35] independently reported similar strategies for the solid phase of small organic molecules in a combinatorial approach concept.

### 1.5. Aryl Hydrazine

**Scheme 6:**
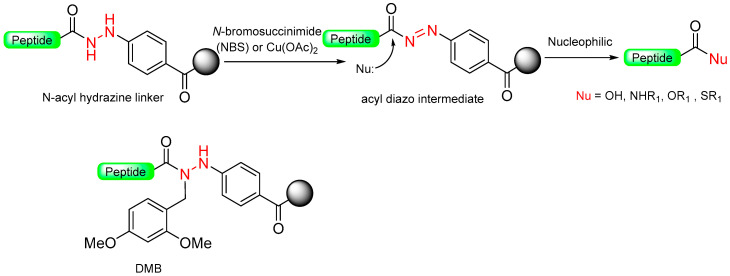
The latent arylhydrazine safety-catch linker.

Wieland et al. [36], based on a Patchornik work [37], developed a safety-catch linker using benzyl hydrazide functionality. An unreactive N-acyl hydrazine linker was transformed into a reactive diazene intermediate through oxidation with N-bromosuccinimide (NBS) (Scheme 6). The acyl diazo intermediate reacts with nucleophiles, amines, or alcohols to produce functionalized acyl derivatives, releasing nitrogen in the process.

Berst et al. developed a better safety-catch linker containing latent aryl hydrazine, designed to be compatible with both Mitsunobu and N-alkylation and stable toward nucleophiles [38]. However, during their investigations, they discovered that the aryl hydrazine safety-catch linker was incompatible with Mitsunobu N-alkylation conditions, leading to the formation of unwanted byproducts through linker alkylation [38]. To address this issue, they employed the 2,4-dimethoxybenzyl (DMB) protecting group to block the reactive hydrazine functionality (Scheme 6). The cleavage of the 2,4-dimethoxybenzyl arylhydrazine (DMBAH) linker from the solid support was achieved in two steps: first, acidic treatment with TFA-DCM was used to remove the DMB protecting group, and then oxidation of the resulting acyl arylhydrazine was carried out in the presence of copper (II) acetate in methanol, serving as an external nucleophile to render the methyl ester [39]. Later, this arylhydrazine linker strategy was used for the synthesis of esters, thioesters for Natural Chemical Ligation (NCL), and p-nitroanilides [40].

Numerous proteins that play essential roles in cell growth and differentiation have ester groups located at their C-terminal ends [41]. Lowe et al. explored various methods for synthesizing peptide methyl esters, a process needed in peptide chemistry [42]. Traditional approaches involved using different resins like Merrifield, 3-thiopropionic acid [43], oxime [44], or hydroxymethyl benzoic acid (HMBA) resin. These methods required specific conditions, such as the presence of tertiary bases, methanol, and sodium methoxide, to cleave the methyl ester from the resin [42]. However, a technique emerged, employing the aryl hydrazide linker [45], which allowed for direct ester generation from the solid support [46]. This innovative strategy utilizes oxidative cleavage in a one-step process, employing copper(II) acetate alongside a suitable nucleophile [47]. Furthermore, NBS can also be employed as an oxidizing agent, proving to be an efficient approach to synthesizing peptide methyl esters [48].

### 1.6. Dihydroquinoline (DHQ)

**Scheme 7:**
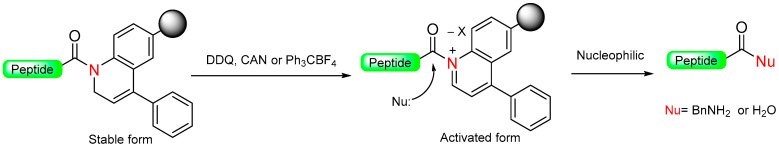
Oxidative aromatization safety-catch linker.

Mioskowski et al. developed a resin-bound compound called 1,2-dihydroquinoline (DHQ) that can generate amides or carboxylic acids peptides (Scheme 7) [49]. The DHQ resin in its *N*-acylated form was proven to be stable even under various conditions, such as basic, acidic, and mild reducing environments. It also proves to be compatible with Boc/Fmoc standard deprotection conditions, although no peptides were synthesized. To activate the linker, oxidative aromatization was performed using substances like 2,3-dichloro-5,6-dicyanobenzoquinone (DDQ), cerium (IV) ammonium nitrate (CAN), or triphenylcarbenium tetrafluoroborate (Ph_3_CBF_4_). This process led to the formation of an activated acyl quinolinium intermediate. The final amides or carboxylic acid peptide was released by displacing the quinolinium moiety using the nucleophiles BnNH_2_ or H_2_O [49].

### 1.7. The Reductive-Acidolytic Safety-Catch Linkers

Although not green at all, TFA is an excellent reagent to remove the Boc group in a Boc/Bzl strategy and carry out the final global deprotection (removal of the side-chain protecting groups and cleavage of the peptide from the resin). In this regard, several linkers have been developed where the lability in front of the TFA is masked during the elongation of the peptide on the resin, and at the end of the synthetic process, the modification of the linker makes it labile to TFA. Most of them are based on the pair methylsulfinylbenzyl/methylthiobenzyl, depicted in Figure 1e,f. As discussed in the introduction, the sulfinyl/sulfoxide linker exhibits stability towards TFA, but it is converted to TFA labile after reduction to the thiobenzyl derivative. However, at the end of synthesis, a reductive treatment converts the sulfinyl/sulfoxide into a thio (Table 1), which is an electron-donating group and, therefore, makes the ester bond cleavable with TFA. Table 1 shows the different reduction methods for C-terminus acid peptides.

**Scheme 8:**
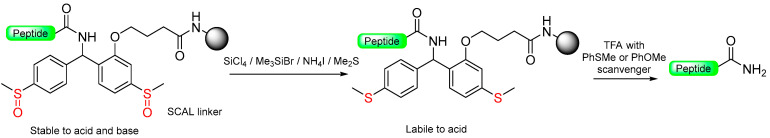
Patek safety-catch acid-labile (SCAL) linker.

Various naturally existing peptides, particularly hormones like oxytocin, secretin, and calcitonin, possess a C-terminal amide function. As a result, Patek and Lebl [63,66] developed safety-catch amide-anchor groups that could be removed using TFA-based methods at the end. Several safety-catch systems have been developed by studying the electronic properties of ortho- and para-substituents in the context of heterolytic benzyl-oxygen or benzyl-nitrogen cleavage dependence [15]. Patek and Lebl introduced a novel safety-catch acid-labile (SCAL) mechanism based on 2-alkoxy-4,4′-bis(methylthio) benzhydrylamine (Scheme 8) [31,67,68]. Additionally, it enables the simultaneous utilization of both Fmoc and Boc groups in peptide synthesis. The oxidized form of the linker, in its sulfoxide state, displays exceptional stability against both acids (such as TFA and thioanisole/TFA) and bases (including aqueous NaOH and piperidine). Activation of the linker occurs through the reduction of both sulfoxide groups to their respective sulfides, achieved either with PPh_3/_Me_3_SiCl/CH_2_Cl_2_ or (EtO)_2_P(S)SH/DMPU [31]. Acidolytic cleavage is subsequently carried out by treating it with TFA in the presence of scavengers, resulting in the formation of C-terminal peptide amides [14,63].

**Figure 2:**
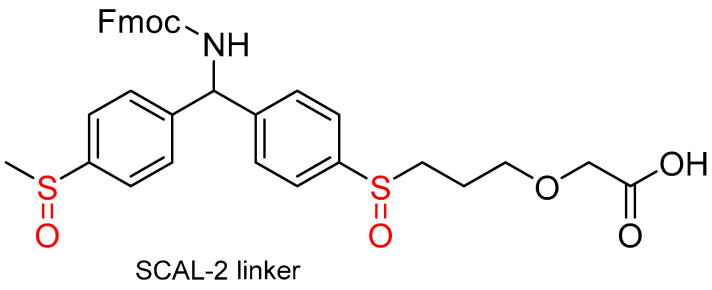
Portal safety-catch acid-labile (SCAL-2) linker.

Portal et al. [69] developed a new safety-catch acid-labile linker (SCAL-2) (Figure 2) with a simplified molecular architecture, easier chemical accessibility, and improved stability compared to the original Patek and Lebl SCAL-1.

**Scheme 9:**
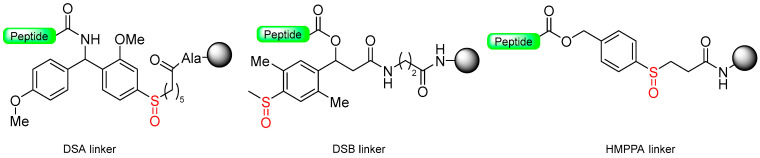
DSA (for amide peptides), and DSA and HMPPA (for acid peptides) as safety-catch linkers.

Kiso and colleagues employed similar strategies in SPPS while designing DSA [61] (4-(4-methoxyphenyl-aminomethyl)-3-methoxyphenylsulfinyl-6-hexanoic acid) and DSB [58] (4-(2,5-dimethyl-4-methylsulfinylphenyl)-4-hydroxybutanoic acid) (Scheme 9). In the DSA linker, amino acids are linked together through an amide bond. This results in the formation of peptide amides once activation occurs, followed by cleavage using TFA [61]. The stability of the DSB linker before reduction was tested using a γ-endorphin peptide comprising 17 amino acid residues. Thus, the peptide resin-DSB-resin was treated with TFA/anisole for 24 h, leading to the cleavage of only 3% of Leu. In contrast, under reductive acidolysis with SiCl_4_-thioanisole-anisole-TFA for 3 h, 94% of Leu was successfully cleaved from the resin [58]. Likewise, Undén and Erlandsson [57] utilized comparable methods in developing the HMPPA (3-(4-hydroxymethylphenylsulfanyl) propanoic acid) linker. The significant application of HMPPA SCLs lies in the synthesis of side-chain-to-side-chain cyclic peptides, as demonstrated by the synthesis of the model peptide Fmoc-K(Boc)FDAPE(OtBu)G-O-HMPPA(O) on aminomethyl resin.

**Scheme 10:**
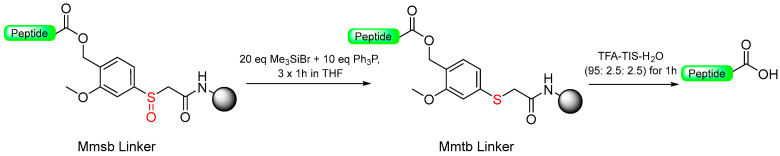
Mmsb safety-catch linker.

Our group introduced the Mmsb linker [13], which is compatible with both Boc and Fmoc chemistry SPPS. Peptide-O-Mmsb-resin was synthesized, and the linker exhibited stability to the treatments, with piperidine and TFA used for the removal of Fmoc and Boc protecting groups, respectively. The peptide can be detached using a two-step protocol. Firstly, the sulfoxide moiety is reduced to the sulfide using Me_3_SiCl and Ph_3_P. Subsequently, treatment with TFA completes the detachment process. The Mmsb linker was applied for the synthesis of somatostatin (Scheme 10).

**Scheme 11:**
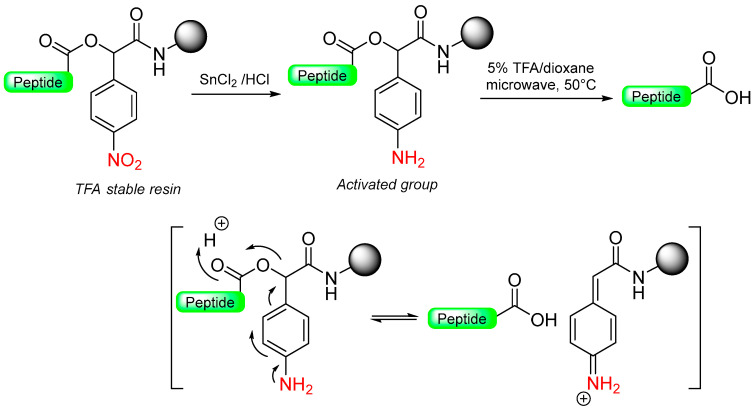
*p*-Nitromandelic-based safety-catch linker.

Our group described a p-nitrobenzyl alcohol (p-nitromandelic acid) linker that forms an ester with the first amino acid stable to TFA and, therefore, is compatible with a Boc strategy but not compatible with Fmoc chemistry [70]. At the end of the elongation of the peptide sequence, the nitro group is reduced very smoothly to amines using SnCl_2_ in HCl/dioxane. The reduced linker liberates the acid peptide via a 1,6-electron pair shift by microwave irradiation at 50 °C with 5% TFA in dioxane (Scheme 11). Cleavage was also attempted in basic media (DIEA), but the yield reported was very low, probably due to the high reactivity of the quinonimine methide generated with nucleophiles [70].

### 1.8. Heterocycle Formation—Via Intramolecular Cyclization—As Leaving Group

The high stability of the five-member ring heterocycles allows their easy preparation by intramolecular cyclization. The presence of an atom of N in the heterocycle makes these heterocycles acidic and, therefore, could be suitable leaving groups, which are the liberation of some acids (acid peptides). This chemical strategy was developed many years ago for the preparation of C-terminus modified peptides, in recent years, the need to prepare a thio ester peptide readily usable in native chemical ligation has emerged [40].

### 1.9. Dpr(Phoc) Linker–Imidazolidinone (Cyclic Urea)

**Scheme 12:**
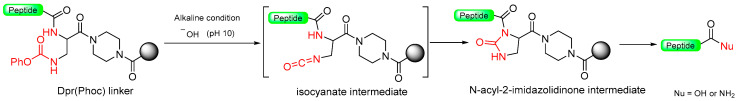
Dpr(Phoc) safety-catch linker.

Pascal et al. [71] introduced the Dpr(Phoc) safety-catch linker (Dpr = L-2,3-diaminopropionic acid, Phoc = Phenyloxycarbonyl), derived from *β*-aminophenyloxycarbonyl-2,3-diaminopropionic acid, which is stable in both basic and acidic conditions [72]. Once the peptide elongation has been concluded, the treatment of the peptide resin under mild alkaline conditions (2 equiv. of 0.04M NaOH for 1 h) renders the electrophilic isocyanate, which suffers an intramolecular cyclization toward the adjacent secondary amide group [73]. Once the N-acyl-2-imidazolidione has been formed, the linker undergoes cleavage through alkaline hydrolysis (0.03M NaOH) or ammonolysis (satd. NH_3_ in iPrOH) or potentially other nucleophiles (Scheme 12). This linker’s stability is notable under various conditions, including 1M in TFA at 0 °C, 50% TFA, and 6 M aq. HCl [74]. The Dpr(Phoc) linker is an aqueous media cleavable linker that can be useful for biological applications of synthetic peptides. It was successfully used to avoid diketopiperazine formation during the synthesis of Tyr-Asp-Pro-Ala-(Pro)6-OH. The Dpr(Phoc) linker can also be applied for the preparation of peptide conjugates and cyclic peptides.

### 1.10. Diamino Benzoic Acid (Dbz)–Benzimidazolinone (Cyclic Urea)

**Scheme 13:**
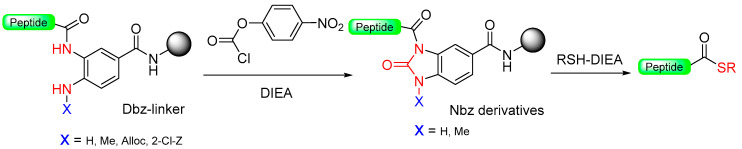
Dbz safety-catch linker.

The generation of C-terminal peptide thioesters constitutes a crucial step in the native chemical ligation method for creating fully or partially synthetic proteins [75]. However, efficiently producing these thioesters through Fmoc solid-phase peptide synthesis proves challenging due to the labile nature of thioester linkage under basic conditions. The functionality of C-terminal N-acylureas (imidazolinones) has been previously investigated in peptide synthesis, as described in the previous section, and similar reports have been made regarding the use of thioacylbenzimidazolinones [76,77]. Blanco-Canosa et al. have developed the 3,4-diamino benzoic acid (Dbz) linker (Scheme 13, X=H) based on that strategy [78]. At the end of peptide elongation, the peptide resin can be chemically modified by treatment with *p*-nitrophenyl chloroformate to yield a *N*-acyl-benzimidazolinone (Nbz) (*N*-acylurea) moiety on the resin. After TFA cleavage of the side-protecting groups, this can cause resin transthioesterification to render the thioester ready to be used in NCL. Alternatively, if the Dbz linker is attached to Rink amide resin, after the cyclization, the *N*-acylurea peptide can be cleaved from the resin with TFA, which can also be used directly for NCL in the presence of a thiol catalyst or transthioesterified.

The important aspect of this approach lies in regulating the chain extension process to ensure that acylation takes place exclusively at one of the two unprotected amines on the Dbz linker (Scheme 13, X = H) [78]. There have been reports indicating that within Gly-rich sequences, acylation of the unprotected o-aminoanilide can take place, particularly in the presence of an excess of bases [79]. The introduction of the protected Fmoc-Dbz (Alloc) effectively eliminates these side-products by employing orthogonal allyloxycarbonyl (Alloc) protection on a single Dbz amine (Scheme 13, X = Alloc) [79]. Additionally, a novel *N*-acylurea linker incorporating an o-amino(methyl)aniline (MeDbz) [40] moiety has been introduced to enhance the robustness of peptide chain assembly (Scheme 13, X = Me). The MeDbz linker has been demonstrated to be superior to the Dbz. It has been utilized in the synthesis of cysteine-rich proteins, such as the cyclotides Kalata B1 and MCoTI-II, through a single cyclization/folding step within the ligation/folding buffer [40]. Furthermore, the MeDbz linker has been used for the on-resin cyclization of side-chain (SH of Cys, NH_2_ of Dab, OH of Tyr)-to-tail cyclic peptides [80,81,82] and for the preparation of C-terminal modified peptides [83,84].

**Scheme 14:**
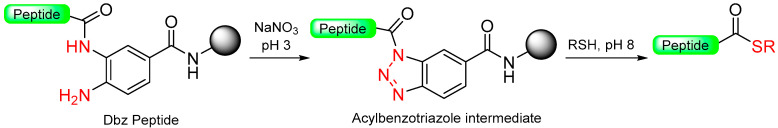
Dbz peptide to thioester peptide via ccylbenzotriazole intermediate.

This concept of using unsymmetrical diamino benzoic resin has been further described by other groups who utilize different protecting groups for the second amine function [75,85,86]. Activation through a peptidyl-benzotriazole intermediate enables easy conversion to peptide thioesters for application in NCL [87]. Using the same Dbz linker, at the end of synthesis, the unprotected peptide can undergo treatment with sodium nitrite, resulting in an acyl benzotriazole intermediate. This intermediate can then be intercepted by a thiol, ultimately producing C-terminal thioester peptides [88] (Scheme 14). A similar linker and strategy were later proposed by Kao et al. [89] for the formation of the benzentriazole isoamylnitrile. Dawson et al. [90] utilized a comparable strategy (formation of the acybenzotriazole) for the in-solution activation of unprotected peptides for further NCL.

### 1.11. Pyroglutamyl/Pyrrolidinone Amide

**Scheme 15:**
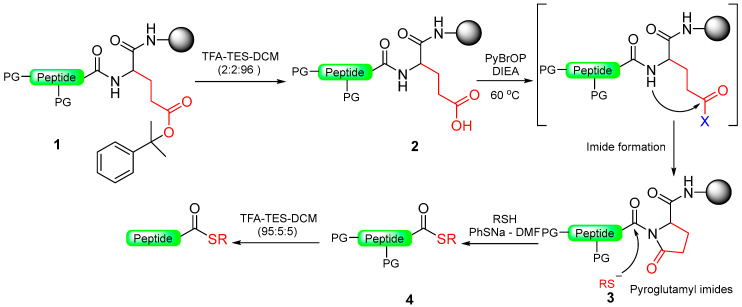
Peptide thioester synthesis through pyroglutamyl imides strategy.

Jensen and co-workers introduced a new method for providing peptide thioesters based on the activation of a backbone amide in the peptide through the formation of a backbone pyroglutamyl imide (Scheme 15) [91].

The synthesis of C-terminal peptide thioesters using a pyroglutamyl imide as a linker strategy is carried out by first attaching a C-terminal glutamic acid residue with a selectively removable side-chain protecting group to a solid support **1**. After the completion of the peptide chain elongation, the Glu side-chain was selectively deprotected **2**. The crucial step involves the activation of the deprotected carboxylic acid with bromo-tris-pyrrolidino-phosphonium hexafluorophosphate (PyBrOP), leading to on-resin formation of the pyroglutamyl (pGlu) imide moiety **3**. The next step was nucleophilic displacement by treatment with thiol (thiolysis) to release the protected peptide thioester **4** from the solid support, followed by the removal of the protecting group in solution (Scheme 15). This linker allows the preparation of thioester peptides with only moderate yields.

**Scheme 16:**
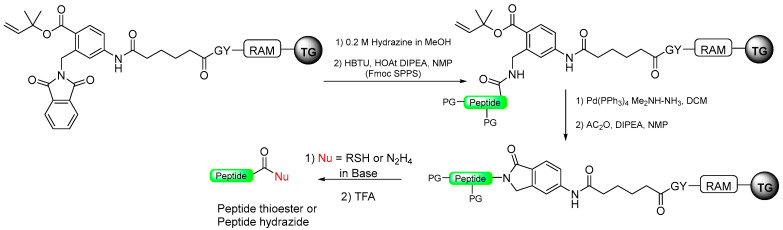
Peptide thioester and hydrazide synthesis through pyrrolidinone strategy (TG-RAM for Tenta Gel RinkAMide).

To address this issue of poor reactivity, Jensen’s group introduced a more reactive aromatic pyrrolidinone resin in a later publication [92]. This resin, based on the same five-member ring principle, exhibits enhanced reactivity in the presence of a nucleophile (Scheme 16).

### 1.12. Cyclic Urethane Moiety

**Scheme 17:**
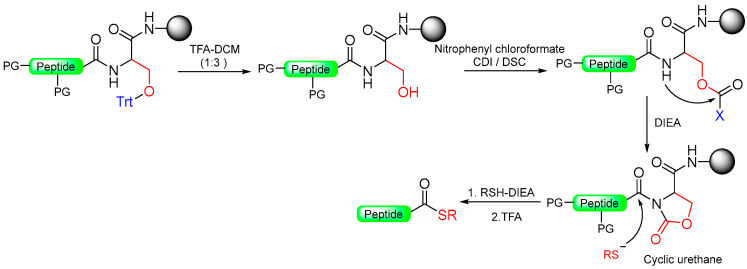
Peptide thioester synthesis through cyclic urethane strategy.

This five-member-ring journey ends with cyclic urethane after cyclic urea and a pyroglutanyl moiety. The method involves the selective activation of the backbone amide bond at the C-terminal Ser residue to generate a cyclic urethane moiety on a solid support [93]. The synthesis process includes anchoring a C-terminal serine residue with a removable side-chain protecting group to the solid support. After peptide chain elongation, Ser’s side-chain is deprotected and activated as an active carbonate with nitrophenyl chloroformate or disuccinidyl carbonate (DSC) or carbonyl diimiazole (CDI), leading to the formation of a cyclic urethane moiety on the resin. Subsequent nucleophilic displacement of the cyclic urethane moiety through treatment with a thiol releases the peptide thioester from the solid support, allowing for subsequent deprotection in solution (Scheme 17) [94]. The thioester produced is free of epimerization and is effectively applied to synthesize large peptides through NCL. (Table 2).

## 2. Conclusions

Safety-catch linkers (SCLs) play an important role in solid-phase peptide synthesis by providing flexibility and stability during the elongation of the peptide chain and allowing safe release of the peptide from the solid support once the linker has been modified. Various types of SCLs have been developed, each with its own application and mechanism of action. The Kenner and Ellamn sulfonamide safety-catch linker, for example, utilizes acyl sulfonamides, which remain stable under various conditions due to their acidic SO_2_NH group. Activation of this linker involves N-methylation, leading to the formation of a labile N-methyl acyl sulfonamide that can be cleaved in the presence of nucleophiles. Similarly, the oxidative safety-catch linkers, introduced by Marshall, involve the oxidation of phenolsulfide resin to produce a sulfone linker, facilitating cleavage through alkali hydrolysis or ammonolysis. However, challenges such as lability in front of bases limit their practical use. Noki linkers overcome all these problems; the peptide linker bond is totally stable to the elongation of the peptide using a Fmoc/tBu strategy, and after oxidation, it liberates the peptide by treatment with secondary amines. This linker, when used in a multidetachable strategy, could be very useful for methodological studies. Other types of SCLs, such as aryl hydrazine and reductive-acidolytic linkers, offer alternative strategies for peptide synthesis, each with its own advantages and limitations. For instance, the aryl hydrazine linker developed by Wieland et al. undergoes oxidation with N-bromosuccinimide to generate a reactive diazene intermediate, allowing for the formation of functionalized acyl derivatives. Moreover, advancements in linker design, such as MeDbz linkers, provide improved stability and compatibility with peptide synthesis strategies like Boc and Fmoc chemistry.

Overall, safety-catch linkers continue to be a significant component in solid-phase peptide synthesis, offering flexibility and efficiency in the construction of peptide libraries and the synthesis of bioactive peptides with diverse functional groups. Due to the importance of the NCL for the chemical synthesis of large peptides and proteins, it is envisaged that other safety-catch linkers will be described, taking advantage of the stability of the corresponding heterocycles. The construction of these has been the driving force for the development of most parts of linkers suitable for the preparation of thioester peptides.

## Figures and Tables

**Table 1:** Reduction and cleavage conditions of sulfoxide to sulfide.

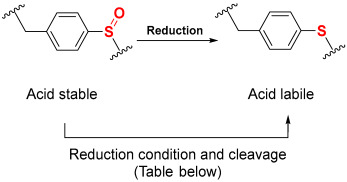		
Entry	Reduction and Cleavage Condition of Sulfoxide to Sulfide	Ref.
1	1.1 eq. of TFAA, 2 eq. of Me_2_S, DCM/THF, 10 min	[50]
2	10 eq. TiCl_4_, 20 eq. of Zn, DCM/THF, 2 h	[51]
3	10 eq. of (Me_3_Si)_2_S, DCM/THF, 4 h	[52]
4	2 eq. Me_3_SiCl/2 eq. PhSSiMe_3_, 1/10 eq. Bu_4_NBr, ether-CHCl_3_, 1 h	[53]
5	10 eq. of Me_3_SiCl, 20 eq. of Me_2_S, THF, 2 h	[54]
6	10 eq. of ZnCl_2_, 20 eq. of Me_2_S, THF, 1 h	[54]
7	20 eq. of Me_3_SiCl, 10 eq. of Ph_3_P, dioxane	[54]
8	10 eq. 1M SiCl_4_/10 eq. TFA-anisole, 10 eq. thioanisole, 10 eq. EDT, 10 min	[55]
9	Me_3_SiBr-EDT in DCM/ACN/DMF/TFA, 15 min-2 h	[56,57]
10	10 eq. SiCl_4,_ 10 eq. thioanisole, 10 eq. anisole, TFA-DCM, 3 h	[58]
11	NH_4_I/Me_2_S/TFA-DCM (2 × 30 min)	[59,60]
12	SiCl_4,_ thioanisole, anisole, EDT, in TFA-DCM, 3 h	[61]
13	Me_3_SiBr/thioanisole, TFA-DCM, 2 h	[62,63,64]
14	20 eq. Me_3_SiCl + 10 eq. Ph_3_P, (3 × 1 h) in THF	[13,65]

**Table 2:** Summary of all the strategies discussed above.

Structures and Names	Linker Modifications	Cleavage Condition	Compatibility with	C-Terminal Release Form	Ref.
**Kenner and Ellman sulfonamide safety-catch linkers**					
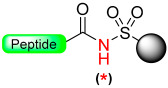 Kenner and Ellman sulphonamide linker	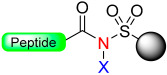 X = CH_3_, CH_2_CN Alkylation with CH_2_N_2_, TMSCHN_2_ or ICH_2_CN Alkylation with Pd (0) Alkylation with *O*, *N*, *N*’- trialkylisoureas	Nucleophiles (hydroxide, amines, hydrazine, thioles)	Boc/Fmoc chemistry	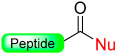 Nu = OH, NHR, NHNHR, SR	[18,19,20,22,23,95,96,97,98,99,100,101,102,103,104]
**The oxidative safety-catch linkers**					
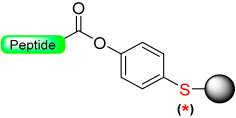	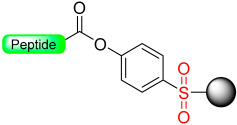 Oxidation with H_2_O_2_ or *m*- CPBA	Nucleophiles (hydroxides, amines)	Boc chemistry	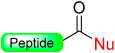 Nu = OR, NHR	[27,28,105]
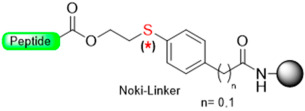	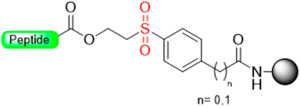 Oxidation with *m*- CPBA	Nucleophiles (hydroxides, amines)	Boc/Fmoc chemistry	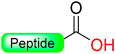	[34,35,106]
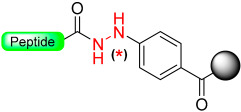	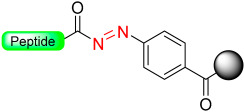 Oxidation with NBS or Cu (OAc)_2_	Nucleophiles (amines, alcohols)	Boc/Fmoc chemistry	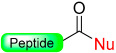 Nu = OR, NHR, SR	[38,39,45,46,47,48]
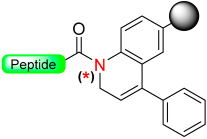	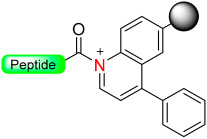 Oxidation with DDQ, CAN or Ph_3_CBF_4_	Nucleophiles (amines, H_2_O)	Boc/Fmoc chemistry	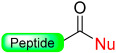 Nu = OPhR, NHR, OH	[49]
**The reductive-acidolytic safety-catch linkers**					
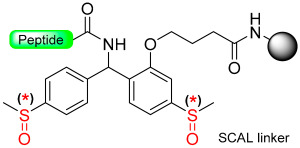	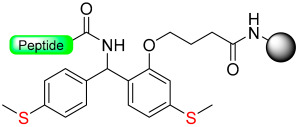 Reduction with SiCl_4/_Me_3_SiBr/NH_4_I/Me_2_S	TFA, with PhSMe or PhOMe scavenger	Boc/Fmoc chemistry	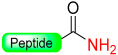	[63,64]
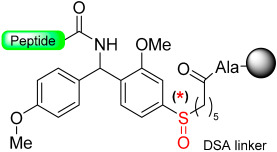	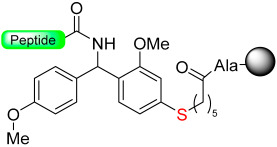 Reduction with SiCl_4_	TFA with PhSMe/ PhOMe)/EDT scavengers	Boc chemistry	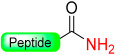	[61]
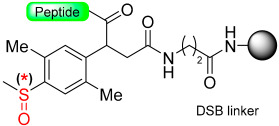	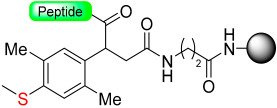 Reduction with SiCl_4_	TFA with PhSMe/PhOMe)/EDT scavengers	Boc chemistry	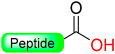	[58]
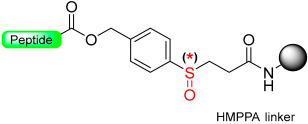	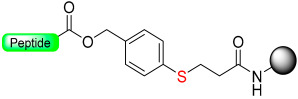 Reduction cleavage with Me_3_SiBr-EDT-TFA	Reduction cleavage Me_3_SiBr-EDT-TFA	Boc/Fmoc chemistry	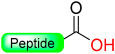	[56,57]
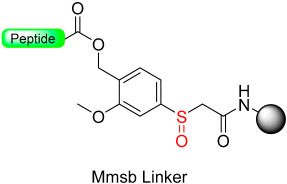	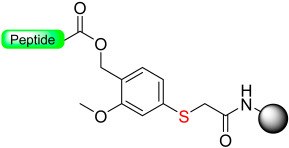 Reduction 20 eq. Me_3_SiBr–10 eq. Ph_3_P-THF	Cleavage TFA: TIS: H_2_O	Boc/Fmoc chemistry	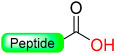	[13]
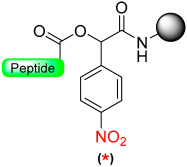	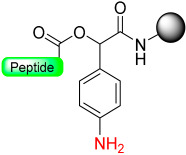 Reduction with SnCl_2_/HCl	TFA with scavengers	Boc/Fmoc chemistry	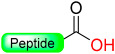	[70]
**Isonitrile/benzamide safety-catch linker**					
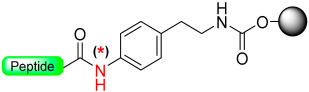	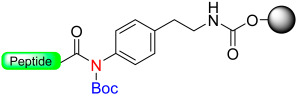 *N*-Protection with Boc	(i) LiOH, 5% H_2_O_2_/H_2_O/THF (ii) MeONa, MeOH: THF	Fmoc chemistry	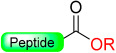 R = H or Me	[26]
Heterocycle formation—via intramolecular cyclization—as leaving group					
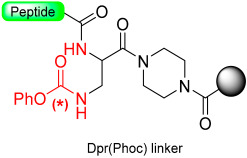	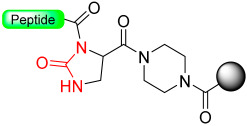 Acylation NaOH to give isocyanate intermediate	Nucleophiles (NaOH or NH_3_ in iPrOH)	Boc chemistry	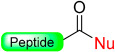 Nu = OH, NHR	[72,73,74]
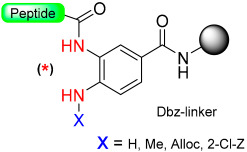	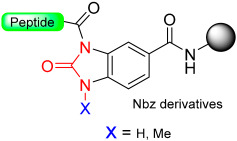 Acylation with *p*-nitrophenyl chloroformate and DIEA to give Nbz derivatives	(i) TFA with TIS scavengers (ii) Thioestherification	Boc chemistry	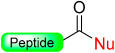 Nu = SR, OR, NHR	[40,78,79,80,81,82,83,84,87,107,108,109]
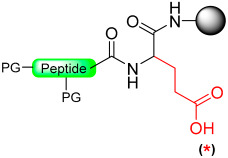	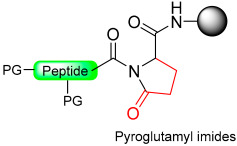 Strong activation with PyBrOP/DIEA	(i) Thiolysis (ii) In-solution cleavage TFA with TES scavengers	Boc chemistry	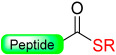	[16,91]
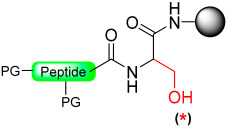	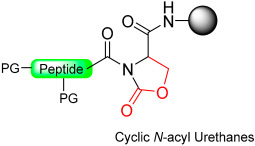 Activation with electrophilic reagent: 4-Nitrophenyl chloroformate/CDI/DSC	(i) Thiolysis (ii) TFA with scavengers	Fmoc chemistry	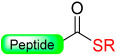	[16,94]
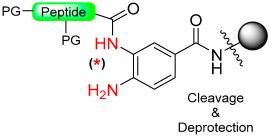	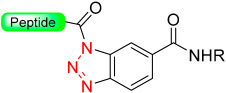 Activation with NaNO_2_ in aqueous buffer	Thiolysis	Fmoc chemistry	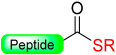	[88]
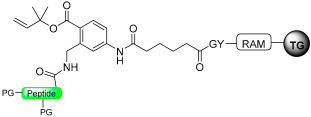	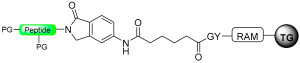 Pd (PPh_3_)_4_ Me_2_NH-NH3, DCM AC_2_O, DIPEA, NMP	Thiolysis Hydrizide TFA	Fmoc chemistry	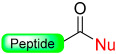 Nu = SR, NHNHR	[92]

**Abbreviations**: ceric ammonium nitrate (**CAN**); diazomethane (**CH_2_N_2_**); triphenylcarbenium tetrafluoroborate (**CPh_3_BF_4_**); 2,3-dichloro-5,6-dicyano-1,4-benzoquinone (**DDQ**); thioanisole (**PhSMe**); anisole (**PhOMe**); trimethylsilyldiazomethane (**TMSCHN_2_**); iodoacetonitrile (**ICH_2_CN**); hydrogen peroxide (**H_2_O_2_**); meta-chloroperoxybenzoic acid (**mCPBA**); *N*-bromosuccinimide (**NBS**); copper (II) acetate [**Cu (OAc)_2_**], 9-Fluorenylation methoxycarbonyl (**Fmoc**); tert-Butyloxycarbonyl (**Boc**); safety-catch acid linker (**SCAL**); silicon tetrachloride (**SiCl_4_**); bromotrimethyl silane (**Me_3_SiBr**); ammonium iodide (**NH_4_I**); dimethyl sulfide (**Me_2_S**); ethanedithiol (**EDT**); hydroxy methyl phenylsulfanyl propanoic acid (**HMPPA**); hydrochloric acid (**HCl**); trifluoroacetic acid (**TFA**); triisopropylsilane (**TIS**); *N*-acyl-benzimidazolinone (**Nbz**); diaminobenzoic acid (**Dbz**); *N*,*N*-diisopropylethylamine (**DIEA**); 2-chlorobenzyl chloroformate (**2-Cl-Z**); bromotris pyrrolidino phosphonium hexafluorophosphate (**PyBrOP**); carbonyl diimidazole (**CDI**); *N*,*N*-disuccinimidyl carbonate (**DSC**).

## Data Availability

Not applicable.
